# Tracing long-term trajectories of contraceptive practice across 185 countries

**DOI:** 10.1371/journal.pone.0205927

**Published:** 2018-10-22

**Authors:** Md Juel Rana, Srinivas Goli

**Affiliations:** Centre for the Study of Regional Development, School of Social Sciences, Jawaharlal Nehru University, New Delhi, India; USC Keck School of Medicine, Institute for Global Health, UNITED STATES

## Abstract

**Background:**

Globally the trajectories of approaches in adoption and implementation of family planning programmes have varied subjecting to variation in cultural and political philosophies across the countries. Accordingly, the progress in family planning has varied over the time across the countries.

**Objective:**

This study investigates long-term trajectories of demand for family planning and contraceptive prevalence rates and tests the hypothesis of convergence across the world countries.

**Methods:**

This study used data from United Nations Population Prospects for 185 countries and regions during 1970–2015. Standard graphical, parametric and nonparametric convergence metrics have been used for testing of the convergence hypothesis.

**Results:**

The results suggest a substantial increase in the global average of both demand and actual prevalence of contraceptive practice across the countries, but the actual contraceptive use is yet to catch up with the demand. Our findings suggest that there is a convergence in contraceptive use across the countries, particularly since the mid-1990s.

**Conclusion:**

A major part of the convergence in demand for family planning and contraceptive prevalence rate is due to its stalling in both developed and many developing countries and its increase in several developing and least developed countries. Family planning has a greater role in human wellbeing particularly enhancing reproductive, maternal and child health outcomes than being a mere tool for fertility reduction. Therefore, the emphasis is needed on family planning efforts in the lagged behind countries for global convergence of family planning.

## Introduction

A large and growing number of studies have identified that the role of family planning is not only limited to fertility decline but also it has its surpassing socioeconomic and health benefits. Family planning directly improves the maternal, newborn, infant and child health through achieving appropriate timing, spacing, and limiting of births [1–6]. It also has indirect effects on women’s education, earnings, employment, and economic benefits through investing on family planning and resultant fertility decline [7–8]. Therefore, the contraceptive use helps women attain their desirable timing, spacing, and limiting of pregnancies and thereby to ensure their reproductive health and rights. Several international commitments have taken place for providing family planning services to those with unmet need for family planning. For instance, Family Planning 2020 (FP2020) targeted to fulfill the demand of an additional 120 million women and girl by 2020 in the highly focused regions of the world. The 7^th^ target of 3rd sustainable development goals (SDGs) is to satisfy the unmet need for family planning of the women at reproductive age.

Before the Second World War, the family planning movement primarily known as a *‘birth control’* movement was led by few personalities through different awareness initiatives. The birth control initiatives were associated with women’s rights to avoid unwanted pregnancies for individual’s wellbeing. Later, during the 1950s and 1960s, a number of studies had come up with their findings that the population control could return economic development [9–11]. After that, a consensus has been made among the international agencies, donors, governments of the USA and some European countries and World Bank to combat the population growth. Since the mid-1960s, the family planning revolution has been initiated and being one of the most controversial public policies; it has experienced a number of upheavals from theoretical to practical grounds in its course. Many countries had embraced their population policies to limit family size and decline fertility in the neo-Malthusian era; later it shifted to the reproductive and right based approach. From the beginning of family planning revolution, due to cultural and political differentials, all the countries had not made their anti-natalist population policy. Later, a vast majority of the countries adopted their population policies, but the political philosophies and implementation strategies had different strength and volume [12]. Therefore, there could be disparities in demand for family planning and their contraceptive use across the regions and countries. In this background, this study investigates long-term trajectories of demand for family planning and subsequent contraceptive use across the region and countries during 1970–2015.

In development studies, to monitor the economic progress of the world countries, convergence or divergence hypothesis is often executed. A convergence hypothesis shows that whether the inequality across the countries is reducing or the lagged nations are catching up with the wealthier counterparts [13–14]. This hypothesis has also widely been investigated for a number of demographic and health indicators, and it has been found that the overall progress does not always ensure the convergence across the countries [15–22]. The journey of the global family planning program has witnessed a trajectory of approaches mainly from Neo-Malthusian to reproductive health and right based approach with wide geographical, cultural and political differentials. In this context, the progress of family planning in individual countries is subjected to the approaches, and therefore, the progress may vary over time. Here, the progress of family planning means the increase of demand for family planning and contraceptive use and the resultant reduction in unmet need. However, the term ‘progress’ has been interchangeably used as ‘positive change’ or ‘increase’ in the paper. The contraceptive use has to catch up the demand to put an end to the unmet need for family planning. However, no study has tested whether global countries are converging or diverging in terms of progress in family planning indicators. Therefore, this study for the first time empirically tests the convergence hypothesis of the progress in demand for family planning and contraceptive prevalence rate (CPR) across the 185 world countries during the recent four and half decades.

## Data and methods

### Data source

This study used data from United Nations Population Prospects estimated by the United Nations Population Division [23]. The dataset provides systematic and comprehensive model-based annual estimates for a range of family planning indicators, including the total demand of contraception, CPR and unmet need pertaining married or in-union women at reproductive age group for 185 countries and regions during 1970–2015. It also provides regional estimates of the developmental regions and continents. Altogether, the estimates have been provided for 42 developed, 47 least developed and 96 other developing countries. The CPR is the satisfied demand for family planning, while the remaining demand is an unmet need. For generating the estimates, a Bayesian hierarchical model was used combining country-specific time trends. The model accounts for the sample characteristics of different data sources and contraceptive methods in various survey-based estimates of prevalence. Details of the methodology of estimation are described elsewhere [24].

### Methods

The long-term trajectories of demand for family planning and CPR at the regional level have been shown in line graphs. For analysing the progress in cross-country trends and the goal of equity, the convergence hypothesis have been tested using three types of convergence metrics. First, the catching up process of the disadvantageous countries towards the advanced countries have been captured using two-way scatter graphical plots. Second, the volume of convergence across the countries has been measured through the standard parametric statistical models such as Sigma (σ) and Beta (*β*) convergence. Third, the Kernel density estimates and plots as a standard non-parametric econometric model of convergence have also been used to show the convergence clubs over the period. The detailed elaboration of the convergence models has been specified below.

### Catching up process

The catching up process indicates that whether laggard countries progressed faster than the advanced countries. It is measured through two-way plot showing change in the contraceptive demand and CPR on ‘y’ axis and their base level on the ‘x’ axis. It does often work as the prerequisite for robust convergence analyses. Although the scatter plots show a glimpse of catching up process, they do not quantify the convergence level. Furthermore, despite showing considerable catching up process in the scatter plots, sometimes, convergence is not ensured, and that divergence can also be experienced [16]. Therefore, robust parametric and non-parametric statistical models have been further used to test the convergence hypothesis.

### Parametric convergence models

Parametric convergence models include σ and *β* convergence. The σ convergence measures the change in inequality across the countries, while the *β* convergence quantifies the progress of laggard nations to the advanced ones [13, 25–27]. Thus, both the methods of convergence needs to be applied to measure the inequality and catching up process across the countries. Always the σ convergence model is supplemented with *β* convergence because the divergence after convergence cannot be captured only with *β* convergence model (Young *et al*., 2008). Thus, the *β* convergence model is necessary but not sufficient for σ convergence. The σ convergence is often measured by standard deviation or coefficient of variation. However, in this study, along with the standard deviation as an absolute measure, the Gini coefficient as a relative measure has also been estimated and presented in graphical form.

Since the *β* convergence model estimates the volume of convergence, it has been applied to analyse whether the demand for family planning and CPR in laggard nations have increased faster than the better-off nations [14, 28]. The absolute *β* convergence refers to a negative association between the annualised growth rate of an indicator and its base value [21]. The following equation denotes the statistical specification of this model:

$$\ln\left[\frac{Y_{i,t+k}}{Y_{i,t}}\right]=\alpha +\beta *\ln\left(y_{i,t}\right)+\varepsilon_{i,t}$$

Where $In\left[\frac{Y_{i,t+k}}{Y_{i,t}}\right]$ denotes annualised growth rate of the variable *Y* in the country *i* in the period (t, t+k), *Y*_*i*.*t*_ is the base value of time *t* and *ε*_*it*_ are the corresponding stochastic term.

The absolute *β* convergence model has been used to estimate the convergence for both the demand for family planning and contraceptive practice, while the conditional *β* convergence model is applied for CPR only. Here, the demand for family planning is the conditional variable for the convergence of CPR across the countries as the demand is the precondition of contraceptive practice. In the statistical model, the conditional variable, demand for family planning is incorporated to control its effect on CPR.

Long-term assessments of convergence may sometimes hide short-term trends in convergence for sub-periods [16]. Hence, for better understanding and from a policy perspective, short-term convergence analysis is useful in long-term trajectories. Therefore, the entire duration of analysis (1970–2015) has been disaggregated into five sub-periods (1970–1975, 1975–1985, 1985–1995, 1995–2005, and 2005–2015).

### Non-parametric convergence estimates

Though parametric convergence measures such as σ and *β* convergence metrics give useful estimates of convergence process, the convergence club in the non-parametric methods offers a useful alternative method for analysing convergence process as it does not assume any assumption about nature of distribution and smoothness of data [26, 29]. Hence, the Kernel density plots have also been used to identify the clusters of demand for family planning and CPR as a measure of the non-parametric test. The kernel estimator can be shown as,

$$\hat{f(x)}=\frac{1}{nh}\sum_{i=1}^{n}K\left(\frac{x_{i}-x}{h}\right)=\frac{1}{nh}\sum_{i=1}^{n}K(Yi)$$

Where, *Y*_*i*_ = *h*^−1^(*x*_*i*_−*x*), *n* denotes the number of countries, *h* is the bandwidth which is a function of the country and tends to zero as n → ∞.

## Results

Prior to showing the results from convergence analyses, the regional perspectives of trajectories in demand for family planning and CPR have been presented as the convergence analyses cannot identify the laggard regions.

### Regional trends and patterns

**Fig 1:**
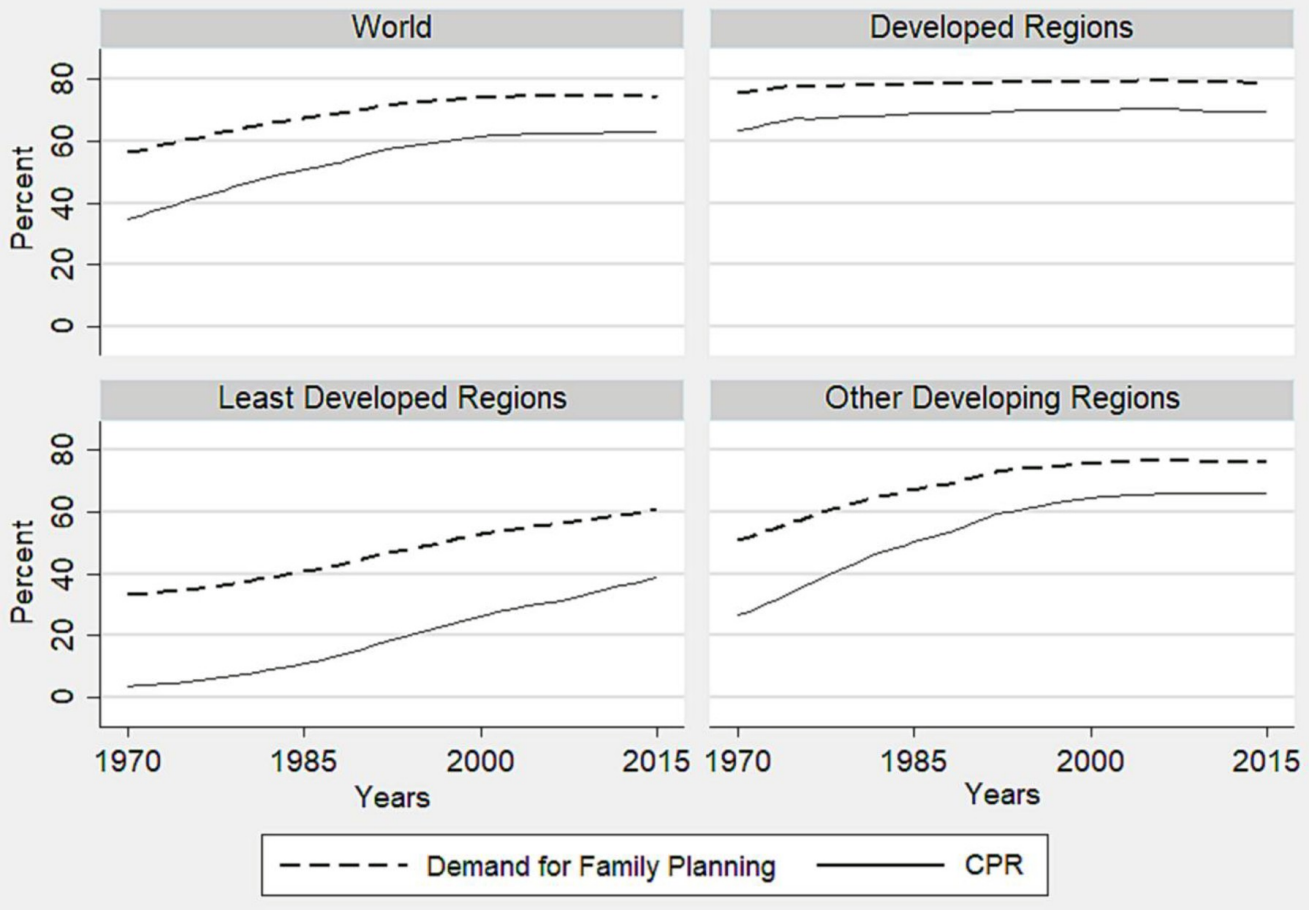
Trends of the demand for family planning and CPR in development regions of the world, 1970–2015.

The trend of demand for family planning and CPR by development regions has been presented in Fig 1. In the study period, both the levels of demand for family planning and CPR in the developed region have always been higher than the other developing and least developed regions. Globally, the demand for family planning has been raised from 56% in 1970 to 74% in 2015, while CPR increased from 35% to 67% during the same time. In the developed regions, the demand and CPR increased from 76% to 79% and 64% to 69% respectively, at the same time, in the least developed region, their improvements were 33% to 61% and 4% to 39% respectively. During the same time, the demand and CPR have been augmented from 51% to 76% and 27% to 66% correspondingly in developing countries. The gap between the demand for family planning and CPR is considered as an unmet need for family planning. Although both demand and CPR considerably increased up till the 1990s, hardly any upward trend has been observed in the previous two decades globally as well as in other developing regions. As a result, the unmet need for family planning declined until the 1990s, but later, a very little positive change has been found worldwide and in the other developing region as well. The developed regions gained little change and remained almost constant in demand for family planning, CPR and unmet need during the study period. In the least developed region, the most disadvantageous one, the take-off in demand and CPR started after the 1980s. The unmet need for family planning in the least developed regions has always been highest, nonetheless, it has begun to decline since the 1980s at a slow pace.

**Fig 2:**
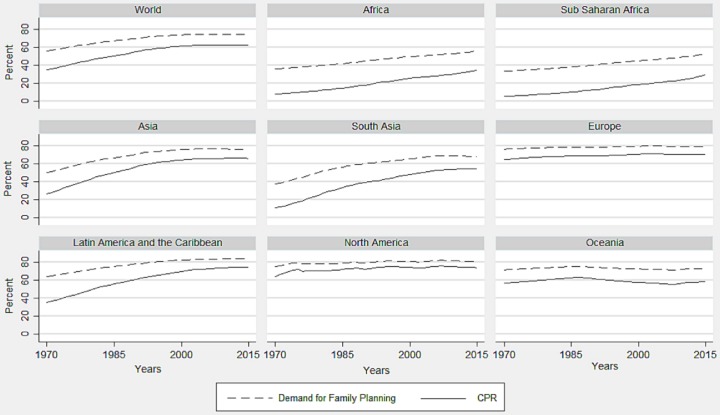
Regional trends of the demand for family planning and CPR, 1970–2015.

Fig 2 demonstrates the geographical region wise trend of demand for family planning and CPR. Apart from the continent wise estimates, two sub-continents such as Sub-Saharan Africa and South Asia have been presented additionally for their voluminous population size and poor performance in terms of family planning indicators. In the developed region such as Europe and North America, the demand for family planning and CPR was highest in the world, while unmet need for family planning was lowest, and all the indicators persisted at the same level during the last four and half decades. In Oceania, including the countries of Micronesia, Polynesia, and Melanesia apart from New Zealand and Australia, both the demand and CPR have been lower and unmet need for family planning was higher than North America and Europe, and they continue to maintain the similar pattern during the study period. The Latin America and the Caribbean increased its demand and CPR, and reduced its unmet need for family planning to the level of developed regions in the recent decades. Although Asia has made a moderate improvement in the previous decades, its performances in terms of family planning indicators have always been poorer than Latin America and the Caribbean. In Asia, both demand for family planning and CPR have been lower and unmet need for family planning has been higher in South Asia than the Asian average. Both the indicators of family planning stagnated since last decade in Asia in general, with only a slight positive change noted in South Asia. Importantly, this stagnation was at a lower level than that of Europe, North America, South America and the Caribbean. Africa, especially the Sub-Saharan region, has been the most disadvantageous region in the world throughout the period. It had lower levels of demand for family planning and CPR, and higher unmet need for family planning in 1970, with slower advancements in the subsequent decades as compared to other regions in the world. The following sections describe the results of country-level convergence analyses.

### Convergence analyses

#### Catching up process

**Fig 3:**
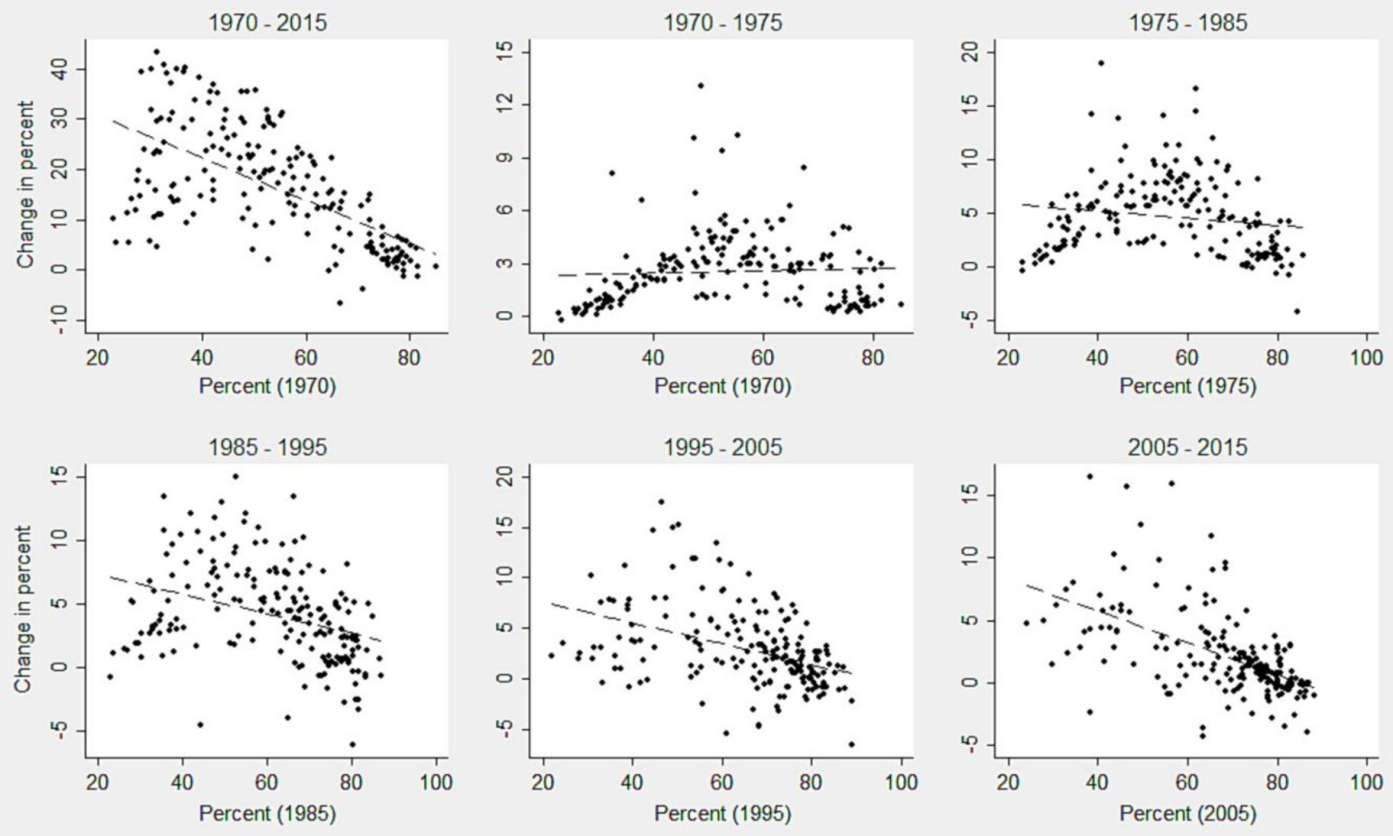
Catching up process of demand for family planning across the countries during 1970–2015.

The scatter plots showing the change in demand for family planning during the respective period on *‘y’* axis and the levels of base years on the *‘x’* axis across the countries are presented in Fig 3. Overall the results for the period 1970–2015 indicated a significant catching-up process. In particular, the first set of states with less demand for family planning at their base year move ahead of the average catching up process. The second set of states with less demand for family planning showed slow progress. The third set of states which had a high demand for family planning at their base year showed stagnation, or only a small increase or even decrease in few cases for subsequent decades. When fragmenting up the total period into smaller periods, diverse patterns in catching up process were observed. During 1970–1975 and 1975–1985, a considerable number of countries with lesser demands for family planning had insignificant catching up process with the advanced nations. Nonetheless, some countries with medium demand had a relatively higher increase during 1975–1985. Along with the countries with medium demand for family planning, those with less demand started to catch up since 1985–1995. After this period, the countries with higher demands for family planning stagnated in catching up process.

**Fig 4:**
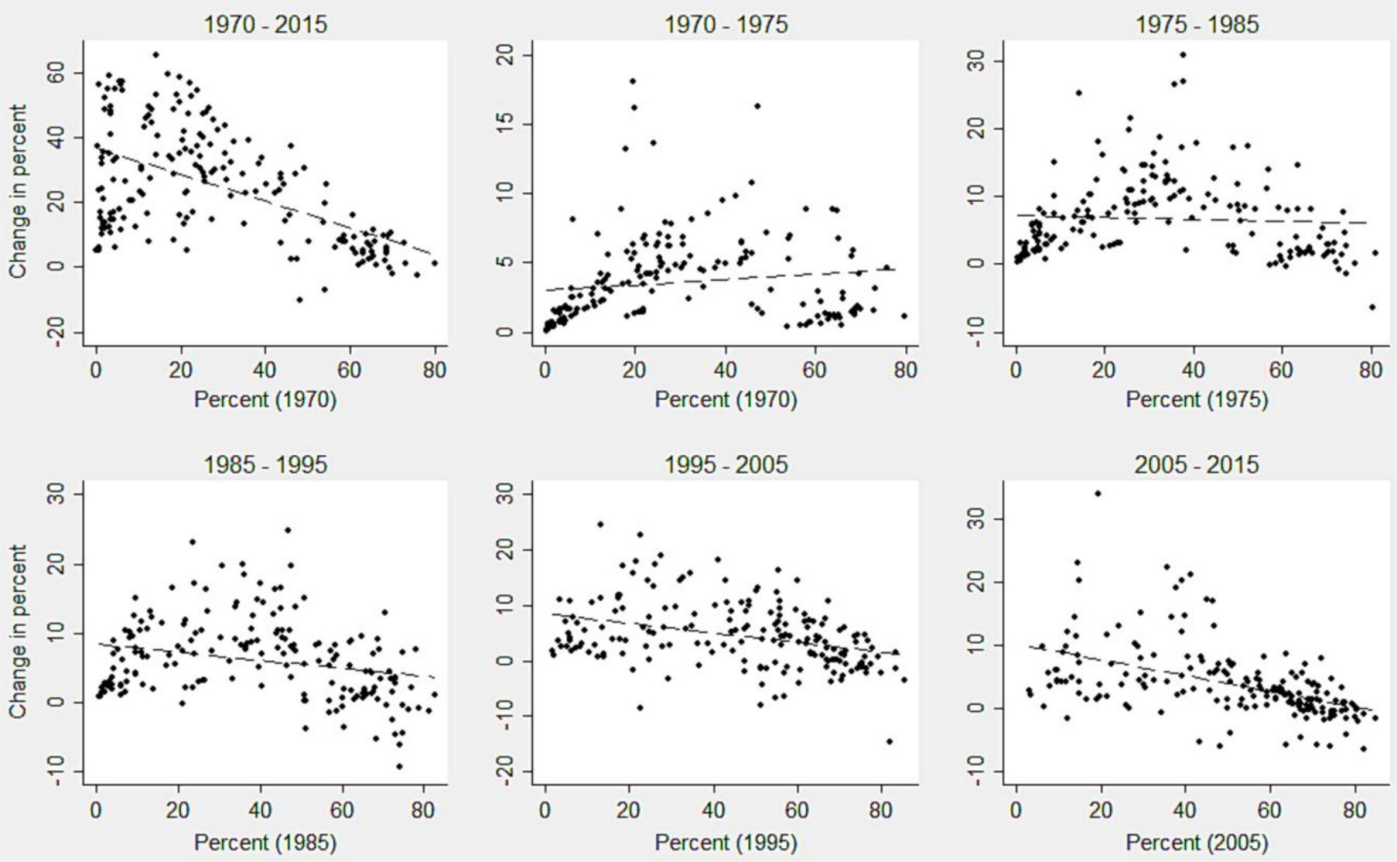
Catching up process of CPR across the countries during 1970–2015.

Fig 4 shows the scatter plots of the change in CPR during the respective years on *‘y’* axis and the levels at the base year on *‘x’* axis across 185 countries. Overall, alike the pattern of catching up process for the demand of family planning, during 1970–2015, the countries with lesser CPR in 1970 showed considerable catch up with the countries having higher CPR. However, except few outliers, the countries can be identified mainly into three groups, first, those with a lesser level of CPR and significant positive change, second, those with a smaller to medium levels of CPR and lower positive change, and third, those with a higher level of CPR and little positive change. During 1970–1975, the change in CPR was for both group of countries, with those having lesser as well as higher levels of CPR at their base years, and thus showing stagnation or slight divergence. Similarly, almost no catching up process was identified during 1975–1985. However, in the following decades, acceleration in catching up process was noted. During the last decade, a set of countries were identified to have lower levels of both CPR at the base year and slow positive growth. Such variations in the catching up process do not always result in the convergence. Therefore, the following section on *β* convergence describes the levels of convergence across the countries at different time points.

#### β convergence

Table 1 shows the results from absolute *β* convergence model of demand for family planning across 185 countries. The overall results reveal that during 1970–2015, the countries with lower demand for family planning progressed towards the countries which had higher demand (*β* = -1.10, *p*<0.001). The results also show that the volume of convergence has been increasing over the decades. It was lowest in 1970–1975 (*β* = -0.22, *p* = 0.199), and improved considerably in subsequent periods: 1975–1985 (*β* = -0.59, *p*<0.001), 1985–1995 (*β* = -1.08, *p*<0.001), 1995–2005 (*β* = -1.34, *p*<0.001) and 2005–2015 (*β* = -1.66, *p*<0.001).

**Table 1:** Absolute *β* convergence of demand for family planning across 185 countries, 1970–2015.

Period	*β* Coefficient	*p* value	Adjusted R^2^
1970–1975	-.2161551	0.199	0.0036
1975–1985	-.5947910	<0.001	0.0880
1985–1995	-1.077379	<0.001	0.2299
1995–2005	-1.342825	<0.001	0.2878
2005–2015	-1.659880	<0.001	0.4431
1970–2015	-1.099561	<0.001	0.5676

Table 2 presents the results from absolute and conditional *β* convergence models of CPR. The results from absolute *β* convergence model show that alike for the demand of family planning, the countries with poorer CPR have shown considerable catch up with those having better-off CPR during 1970–2015 (*β* = -1.53, *p*<0.001). The degree of progress was lowest in 1970–1975 (*β* = -1.52, *p*<0.001) and increased in subsequent decades: 1975–1985 (*β* = -1.65, *p*<0.001), 1985–1995 (*β* = -2.02, *p*<0.001), 1995–2005 (*β* = -2.17, *p*<0.001), 2005–2015 (*β* = -2.24, *p*<0.001). The results from the conditional *β* convergence model of CPR controlling the demand for family planning indicate that similar to the results of absolute *β* convergence model, the countries with lower CPR significantly advanced towards those who have a better CPR during 1970–2015 (*β* = -1.80, *p*<0.001). Again, the magnitude of catching up process is lowest in 1970–1975 (*β* = -1.05, *p*<0.001), followed by 1975–1985 (*β* = -1.39, *p*<0.001), 1985–1995 (*β* = -1.96, *p*<0.001), 1995–2005 (*β* = -3.70, *p*<0.001), 2005–2015 (*β* = -4.15, *p*<0.001). Interestingly, the *β*-coefficients in conditional *β* convergence model are higher than that of absolute *β* convergence model during 1995–2015 and opposite during 1970–1995.

**Table 2:** Absolute and conditional *β* convergence of CPR across 185 countries, 1970–2015.

Period	*β* Coefficient	*p* value	Adjusted R^2^
*Absolute β convergence*			
1970–1975	-1.517464	<0.001	0.4688
1975–1985	-1.650760	<0.001	0.5984
1985–1995	-2.017723	<0.001	0.6558
1995–2005	-2.168667	<0.001	0.5373
2005–2015	-2.238752	<0.001	0.5413
1970–2015	-1.532050	<0.001	0.8643
*Conditional β convergence*			
1970–1975	-1.047012	0.003	0.4720
1975–1985	-1.391889	<0.001	0.5978
1985–1995	-1.962651	<0.001	0.6540
1995–2005	-3.697183	<0.001	0.5568
2005–2015	-4.154113	<0.001	0.5688
1970–2015	-1.797213	<0.001	0.8671

Note: Conditional *β* convergence estimation is adjusted for the demand for family planning

#### σ convergence

**Fig 5:**
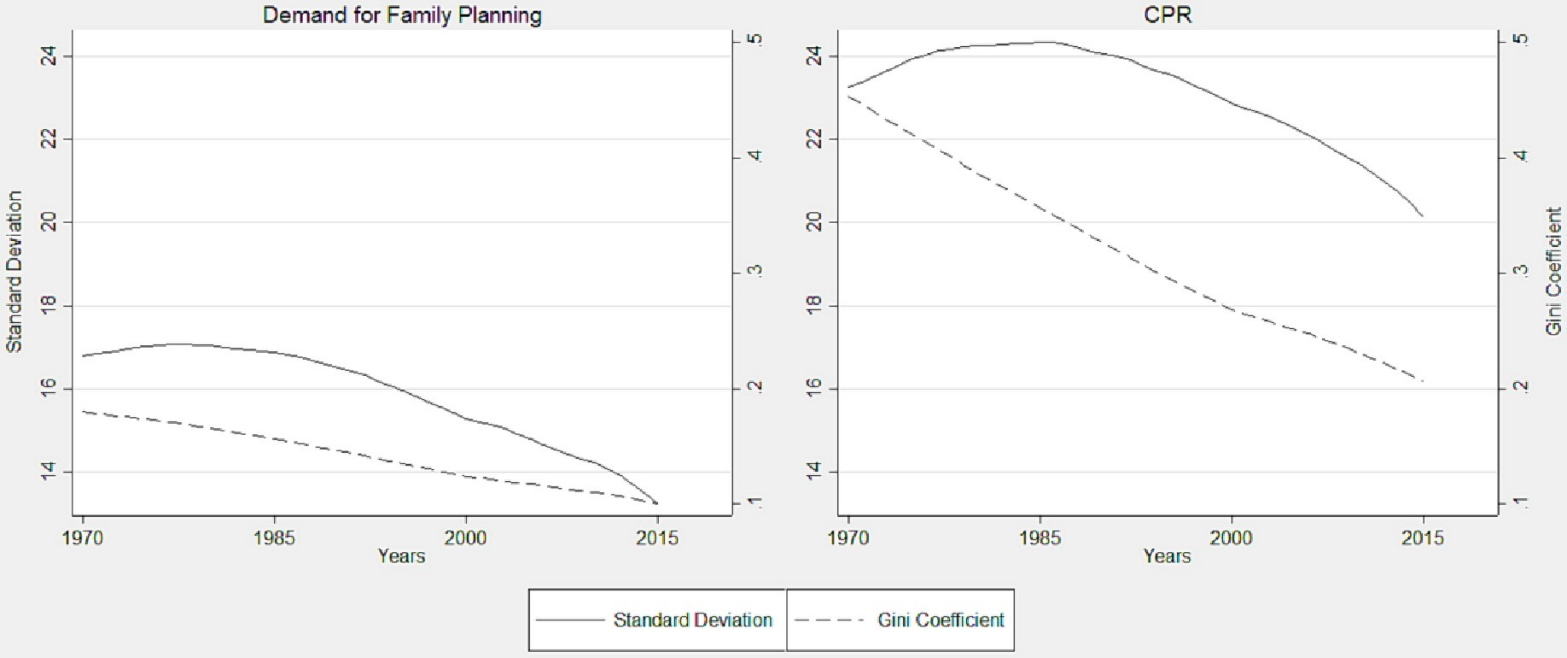
Standard deviation and Gini coefficient across the countries, 1970–2015.

σ convergence estimates show the increase or decrease in inequality in demand for family planning and CPR across the countries. Fig 5 shows two estimates of σ convergence namely standard deviation as an absolute measure and Gini coefficient as a relative measure. The results suggest that overall inequality in demand for family planning was monotonically lower than that of CPR during the study period. The Gini coefficient estimates exhibit that the inequality in terms of both demands for family planning and CPR have been declining, but the decline in CPR was faster than that of the demand. The trend of standard deviation reveals that the inequality in the demand was constant in the 1970s and early 1980s, and further started declining, while the inequality in CPR slightly increased in the 1980s and decreased relatively faster than that of the demand thereafter.

#### Convergence clubs

**Fig 6:**
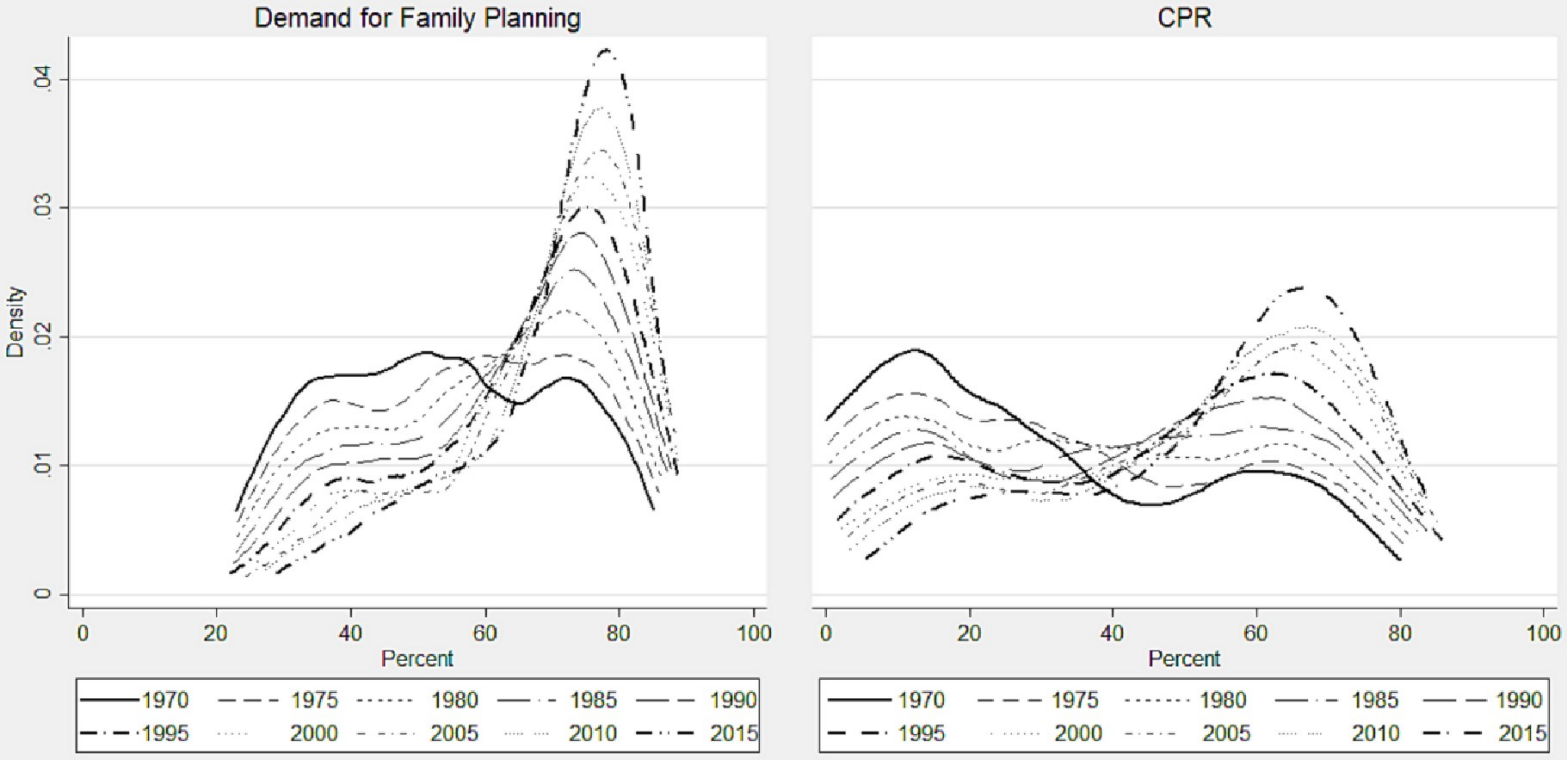
Kernel density plots showing the convergence clubs of the countries, 1970–2015.

Fig 6 displays the Kernel density plots showing the demand for family planning and CPR across 185 countries during 1970–2015 at the interval of five years. The plots indicate that the demand for family planning had two peaks in 1970: the first one is the broad plateau-shaped between 30% and 60%, while the second is the narrow one ranges between 70% and 80%. These bimodal peaks are frequently known as *‘convergence clubs’* in the literature [26]. These peaks signify the divergence across the countries. Over the period, the first peak steadily lowered down and the second one is upsurged. In the recent years, the first peak nearly disappeared and the second peak got its highest level, which indicates convergence among the countries, while a few countries still remain near the first peak indicating that an absolute convergence is yet to be achieved. Similar to the pattern of convergence for the demand of family planning, the CPR had two convergence clubs, however unlikely to the same, its first club had a higher height between 5% and 20% of CPR and the second one had a lower height between 55% and 70% of CPR in 1970. Although the first convergence club consistently declined and the second club concomitantly rose of over the period, a considerable number of countries were still trapped in the first club. This trend indicates that the laggard nations need significant attention for global convergence in family planning.

## Discussion

A number of studies have assessed the global progress of family planning in general but have often been limited to their examination of trends, patterns and determinants at aggregate and local levels [30–35]. However, the convergence or divergence in demand for family planning and contraceptive practice across the countries has not been attempted to be studied so far, particularly, in the context of global population policy shift. In this study, along with the analysis of long-term trajectories from a regional perspective in demand for family planning and CPR during last four and half decades, the convergence hypothesis has also been tested across 185 countries for the period. This study provides some cross-cutting fresh insights on long-term trajectories in demand for family planning and CPR with a regional perspective using standard parametric and non-parametric measures of convergence hypothesis.

### Summary of findings

Findings suggest that both demand for family planning and CPR have stalled at 75%-80% and 70%-75% respectively in developed regions, displaying persistent unmet need for family planning. However, the developed regions have had highest and constant demand for family planning and CPR with lowest and persistent unmet need in the world throughout the previous decades. In a population, the demand cannot be 100% because of infertility, sexual inactivity, current pregnancy and intention of being pregnant, but the CPR can be equal to the demand for family planning if there is no unmet need for contraception. So, though the pattern in developed regions indicate that they have reached their saturation level of the demand for family planning and CPR, there is still scope for reducing the unmet need for family planning by increasing CPR to the levels of their demand for family planning.

In the other developing regions, the levels of both demand for family planning and CPR were higher as compared to the least developed areas in 1970. The other developing regions made significant positive growth until the 1990s, and the growth slowed down thereafter. Contrastingly, the least developed regions observed accelerated growth after the 1990s. However, the present demand for family planning and CPR in the other developing regions are higher than that of the least developed ones. Further, the unmet need for family planning is considerably higher in the least developed regions than the other developing regions which means that the least developed countries even cannot fulfill the lower levels of demand for family planning. Also, the coefficients of the conditional *β* convergence model of CPR were found to be higher than those of the absolute *β* convergence model in the last two decades. This trend of convergence of CPR indicates that the unmet need for family planning has altogether been reducing over the period because the demand for family planning has stagnated over last two decades when the CPR has been catching up to the demand for the family planning.

Broadly, the world countries experienced an increase in both demand for family planning and CPR over the decades, which comprises earlier divergence or stagnation during 1970–1985 and emerging convergence in the subsequent decades. The catching up plots indicate that for both of the indicators, there was significant progress during 1970–2015, but very slow growth or stagnation during 1970–1985. This pattern reveals that the countries with medium levels in demand for family planning and CPR made greater progress than those with the low levels during 1970–1985. During the same period, we observe that the African, especially Sub-Saharan African region, had the lowest levels of both demand for family planning and CPR, and they had a poor growth during subsequent decades as well. The current progress in the convergence across the countries is because of two main mechanisms: firstly, the positive growth of demand for family planning and the CPR stagnated in developed countries, and secondly, their substantial positive changes were registered in the Latin American and Caribbean countries. Although the Asian countries advanced substantially, the South Asian nations showed stalling at lower levels. Since 1995, the acceleration in the convergence of CPR after controlling the demand for family planning was largely led by the reduction of unmet need especially in the Latin American and Caribbean countries and also Asian countries to some extent. The convergence clubs demonstrate that while the progress in demand for family planning is lagged behind for only a few nations, a considerable number of countries are yet to make improvements in the CPR. Such laggard countries are mainly from the African and Asian regions, particularly Sub-Saharan Africa and South Asia.

### Trajectories in convergence: Plausible explanations

The long-term trajectories in convergence are the results of the family planning programs adopted across the countries over the past several decades. The family planning revolution has started since the 1960s. Its advocacy culminated at the International Conference on Population and Development in Bucharest, 1974. However, the wave did not influence the countries evenly as variations were observed across the regions and countries in both demands for family planning and CPR. North America, Europe and Oceania (excluding Polynesia, Micronesia, and Melanesian countries) had constant higher demands for family planning and CPR, with lower levels of the unmet need throughout the past decades compared to the other regions. The higher levels of demand for family planning and CPR in the 1970s for Latin American, Caribbean and Asian countries and considerable increase in subsequent decades, unlike for the African nations, indicates that most of the countries in these regions had participated in the family planning revolution despite their existent cultural barriers. For African countries, their slow progress and late catching up process implies delayed adoption of family planning program, possibly due to cultural and political impediments in poor socioeconomic development settings [12].

During the beginning of the family planning revolution, deliberations were made on political decisions to embrace family planning policies considering many positive and negative aspects. The positive factors were Neo-Malthusianism on economic ground, the satisfaction of unmet need on humanitarian ground and post Second World War baby boom, while the negative aspects included traditional cultures, religious constraints, high child mortality and Marxist view [12]. So, the political commitments across the countries resulted in the variations regarding the adoption of government family planning programs and consequential differences in demand for family planning and CPR across the regions and countries. In 1976, around two-thirds of the total countries (63%) supported family planning program, while 20 governments provided indirect support through the private sectors or non-governmental organizations and the rest 17 states mostly from Africa did not endorse any family planning program mainly due to political and cultural constraints [36]. However, the disparity in political commitments towards adopting family planning programs is still present across the countries. For instance, in 2013, 160 out of 197 governments (81%) directly supported the family planning programs. Notably, in the less developed regions, the proportion of countries supporting family planning programs increased from 82% in 1996 to 93% in 2013. Contrarily, the proportion of governments providing direct support to family planning in more developed regions declined from 1996 (58%) to 2005 (45%), with a slight increase in 2013.

### Policy implications

A steady decline in the number of countries of more developed regions giving direct support for family planning is likely a response to constant low levels of fertility or the consideration that the private sector can supply contraceptives, rendering government support less essential. Over the last several decades, the fertility considerably declined globally, even in the developing countries where the fertility decline is underway. As the concern of population explosion has diminished, the family planning program has lost its importance to reduce the fertility [37–38]. With the largest share of the population of Asia, Africa, and Latin America, they will continue to increase the size of global population even when their fertility reaches the replacement level because of increased lifespans and population momentum [39]. The evidences from our study show that a substantial share of women have unmet need for family planning even in the developed countries. Even after two decades of the International Conference on Population and Development (ICPD), Cairo (1994) commitments regarding protection of reproductive health and rights, an estimated nearly 40% pregnancies of the world are unintended [40]. Further, globally out of about 85 million unintended pregnancies, half of them turned into abortions, 13% were terminated in miscarriages, and 38% resulted in unintended births in 2012 [41]. During 2010–2014, nearly 56 million induced abortions took place each year, with higher incidences of abortion in the developing countries than the developed ones [42]. This indicates that globally a vast share of women are in unmet need for family planning needing immediate consolidated attention through reinvestments on family planning program with a special focus in the developing and least developed regions.

At the beginning of the 21^st^ century, the investment in the family planning program considerably declined [39, 43]. In this background, FP2020 initiative has targeted to provide modern contraceptives to an additional 120 million women with unmet need for family planning in 69 focused resource-poor laggard countries making a global partnership with governments, civil society, multilateral organisations, donors, private sectors and research and development community. The initiatives under the FP2020 have completely been shifted from the Neo-Malthusian to the reproductive right based approach, however, the investments on family planning under the umbrella of FP2020 are criticised for targeting the poor to reduce their number [44]. Such criticism limits the importance of family planning program to fertility decline, and overlooks the surpassing direct and indirect returns of family planning on health, education and economy [1–3, 6]. The modern contraceptive methods cause a genuine *‘reproductive revolution’* and are considered as *‘social vaccine’* [45]. Therefore, as this study shows that the developing and least developed countries in general, and African and Sub-Saharan countries, in particular, have lagged behind in reducing the unmet need for family planning, these backbenchers need special attention in the provision of contraceptive methods to achieve the SDGs by 2030.

### Limitations

This study used a model based estimated database which took different sources of information from the respective countries. The data based on the sources from the developing countries with poorly performing health information systems may suffer from reporting errors. Further, the study could not take account of the roles of demands for family planning and contraceptive practices among the marginalised groups such as adolescent, socioeconomically and physically disadvantageous population in the process of convergence, because of unavailability of disaggregated data. Also, the study could not take into consideration the quality of care of contraception. The rise in CPR does not ensure the quality of care, and may vary across different regional and socio-political settings. Additionally, the improved quality of care may even enhance contraceptive practices. Further research can explore the different dimensions of quality of care in the analyses of trend, pattern, and convergence.

## Conclusion

Our findings show that the levels of demand for family planning across the countries have significantly increased, but growth in CPR is yet to catch up. So, the substantial unmet need for family planning is the matter of concern along with the improvement of the demand for family planning for a group of countries especially Sub-Saharan Africa. Further, even though all the states have been progressing in terms of both demand for family planning and contraceptive practice, the vast gap between the countries especially developed and least developed countries need to be addressed with equity. The recent decline in the international funding raise concerns about the convergence of family planning in developing countries [46–47]. Apart from international efforts, country-specific investments and interventions for fulfilling the unmet need are needed to improve the contraceptive use for achieving the unprecedented SDG family planning goals by 2030 and thereby attain convergence across the countries. The agenda of family planning should sustainably be to ensure *‘every pregnancy*, *a planned pregnancy’* even in the post-SDG era for the coming generations. Further, this convergence analysis can be used as an instrument for measuring and monitoring the equitable and sustainable progress of demand for family planning and contraceptive practice. The effort towards continuous tracking of the progress of the family planning indicators and their convergence at the global and regional level at least five years intervals is required for better monitoring.
